# Supplementary material for: Long COVID at Different Altitudes: A Countrywide Epidemiological Analysis
Source: Int J Environ Res Public Health. 2022 Nov 8;19(22):14673. doi: 10.3390/ijerph192214673 (PMC9690364; doi:10.3390/ijerph192214673)
Supplement: Supplementary file 1 [file ijerph-19-14673-s001.zip › ijerph-1896361-supplementary.pdf]

Supplementary file S1. Demographic characteristics and history of participants with Long-COVID symptoms.

Characteristics		n	(%)
**Demographics**			
Sex	Male	396	36.0%
	Female	704	64.0%
	Total	1100	100.0%
Age (years)	0 to 10	1	0.1%
	10 to 20	60	5.5%
	21 to 30	382	34.7%
	31 to 40	372	33.8%
	41 to 50	191	17.4%
	51 to 60	72	6.5%
	61 to 70	15	1.4%
	71 to 80	6	0.5%
	81 to 90	1	0.1%
	Total	1100	100.0%
Ethnicity	Mestizo	1008	91.6%
	White	59	5.4%
	Montubio	15	1.4%
	Indigenous	8	0.7%
	Afro descendants	6	0.5%
	Other	4	0.4%
	Total	1100	100.0%
Marital status	Single	587	53.4%
	Married	405	36.8%
	Divorced	62	5.6%
	Common-law	41	3.7%
	Widowed	5	0.5%
	Total	1100	100.0%
Residence altitude	Above 2500 m.	789	71.7%
	Below 2500 m.	311	28.3%
	Total	1100	100.0%
Occupation	Health care workers	238	21.6%
	Indoor workers	291	26.5%
	Outdoor workers	14	1.3%
	Workers in contact with people	324	29.5%
	Students	184	16.7%
	Unemployment and retired	49	4.5%
	Total	1100	100.0%
Weight (kg)*		66	(47 - 85)
Height (cm)*		163	(151 - 175)
BMI (kg/cm^2)*		24.8	(19.4 - 30.2)
BMI (kg/cm^2)	Underweight (< 18.5)	29	2.5%
	Normal (18.5 - 24.9)	544	49.5%
	Overweight (25.0 - 29.9)	369	33.6%
	Obesity (> 30.0)	158	14.4%
	Total	1100	100.0%

**Personal history**			
Comorbidities history	Yes	247	22.5%
	No	853	77.5%
	Total	1100	100.0%
Comorbidities	Arterial hypertension	66	25.1%
	Overweight	50	19.0%
	Obesity	28	10.6%
	Diabetes type 2	16	6.1%
	Asthma	27	10.3%
	HIV/AIDS	6	2.3%
	Type 1 diabetes	6	2.3%
	Hypothyroidism	43	16.3%
	Cancer	9	3.4%
	Coagulation disorders	6	2.3%
	Hyperthyroidism	6	2.3%
	Total	263	100.0%
Smoke	No	853	77.5%
	Old smoker	140	12.7%
	Yes, less 5 cigarettes a day	92	8.4%
	Yes, between 6 and 20 cigarettes per day	15	1.4%
	Total	1100	100.0%
Alcohol	No	298	27.1%
	Once every 3 months	441	40.1%
	At least once a month	267	24.3%
	At least once a week	86	7.8%
	More than two times a week	8	0.7%
	Total	1100	100.0%
**COVID-19 infection history**			
Number of infections	Once	827	75.2%
	Twice	239	21.7%
	More than twice	34	3.1%
	Total	1100	100.0%
Diagnostic confirmation	PCR, antigen, or antibody tests	923	83.9%
	Physician confirmation	36	3.3%
	Infected by someone who lived with me	141	12.8%
	Total	1100	100.0%
Clinical management	None	86	7.8%
	Self-medication	160	14.5%
	Medications prescribed by a physician	810	73.6%
	Hospitalization less than 3 days	7	0.6%
	Hospitalization between 3 and 7 days	12	1.1%
	Hospitalization more than 7 days	14	1.3%
	Intensive care unit (ICU) hospitalization	11	1.0%
	Total	1100	100.0%
COVID-19 Vaccination history	No	1	0.1%
	One dose	3	0.3%
	Two doses	173	15.7%
	Two doses and one booster	781	71.0%

	Two doses and two boosters	142	12.9%
	Total	1100	100.0%
Time of infection	Infection before vaccination	561	51.0%
	Infection after vaccination	539	49.0%
	Total	1100	100.0%
**Long-term COVID-19 symptoms**			
Onset symptoms	Initiated with infection	449	40.8%
	3 to 4 weeks after infection	414	37.6%
	5 to 7 weeks after infection	103	9.4%
	8 to 9 weeks after infection	50	4.5%
	Later than 10 weeks post-infection	84	7.6%
	Total	1100	100.0%
Symptoms duration	Between 1 to 4 weeks	224	20.4%
	Between 4 to 8 weeks	182	16.5%
	Between 8 to 12 weeks	115	10.5%
	Between 3 to 6 months	210	19.1%
	Between 6 to 12 months	131	11.9%
	For more than 13 months	238	21.6%
	Total	1100	100.0%
Frequency of presentation	Once a month	97	8.8%
	Once every two weeks	84	7.6%
	Once a week	216	19.6%
	Over 3 days a week	297	27.0%
	Daily	331	30.1%
	Other	75	6.8%
	Total	1100	100.0%
Symptoms evolution	Has improved	389	35.4%
	Has been consistent	345	31.4%
	Intermittent (appear - disappear)	316	28.7%
	Has gotten worse	50	4.5%
	Total	1100	100.0%
Visited a physician	Yes	492	44.7%
	No	608	55.3%
	Total	1100	100.0%
Medications prescribed by a physician	Yes	335	30.5%
	No	765	69.5%
	Total	1100	100.0%
Self-medication	Yes	367	33.4%
	No	733	66.6%
	Total	1100	100.0%

*: Median and interquartile range (IQR), BMI: body mass index, PCR: polymerase chain reaction.

Supplementary file S2. Self-reported long-COVID symptoms distributed by characteristics of Ecuadorian participants.

Symptoms	Total n	Sex n (%)			Comorbidities n (%)			Ethnicity n (%)							BMI n (%)				
		Male	Female	p Value	No	Yes	p Value	Afro	White	Indigenous	Mestizo	Montubios	Other	p Value	Underweight	Normal	Overweight	Obesity	p Value
Alopecia	32	12 (37.5)	20 (62.5)	0.999	22 (68.8)	10 (31.2)	0.237	--	1 (3.1)	--	31 (96.9)	--	--	0.832	1 (3.1)	16 (50)	10 (31.2)	5 (15.6)	0.989
Taste dysfunction	199	56 (28.1)	143 (71.9)	**0.013**	162 (81.4)	37 (18.6)	0.354	2 (1.0)	10 (5.0)	4 (2.0)	178 (89.5)	4 (2.0)	1 (0.5)	0.186	7 (3.5)	118 (59.3)	52 (26.1)	22 (11.1)	**0.01**
Alterations in glucose	16	7 (43.7)	9 (56.3)	0.697	8 (50.0)	8 (50.0)	**0.011**	1 (6.3)	--	1 (6.3)	13 (81.2)	1 (6.2)	--	**<0.001**		4 (25)	10 (62.5)	2 (12.5)	0.052
Menstrual cycle alterations	118	0 (0.0)	118 (100.0)	**NA**	88 (74.6)	30 (25.4)	0.295	1 (0.9)	6 (5.1)	1 (0.8)	106 (89.8)	4 (3.4)	--	0.369	3 (2.5)	63 (53.4)	37 (31.4)	15 (12.7)	0.835
Hallucinations	20	9 (45.0)	11 (55.0)	0.541	16 (80.0)	4 (20.0)	0.999	--	--	1 (5.0)	18 (90.0)	--	1 (5.0)	**<0.001**		9 (45)	9 (45)	2 (10)	0.576
Anxiety	379	111 (29.3)	268 (70.7)	**<0.001**	293 (77.3)	86 (22.7)	0.449	1 (0.3)	15 (4.0)	2 (0.5)	356 (93.9)	3 (0.8)	2 (0.5)	0.365	13 (3.4)	189 (49.9)	119 (31.4)	58 (15.3)	0.466
Brittle hair	111	9 (8.1)	102 (91.9)	**<0.001**	81 (73.0)	30 (27.0)	0.149	--	3 (2.7)	--	105 (94.6)	3 (2.7)	--	0.193	4 (3.6)	59 (53.2)	34 (30.6)	14 (12.6)	0.716
Mood changes	234	80 (34.2)	154 (65.8)	0.566	184 (78.6)	50 (21.4)	0.999	1 (0.4)	19 (8.1)	2 (0.9)	205 (87.6)	5 (2.1)	2 (0.9)	0.148	7 (3)	117 (50)	83 (35.5)	27 (11.5)	0.543
Tachycardia	184	71 (38.6)	113 (61.4)	0.473	142 (77.2)	42 (22.8)	0.641	1 (0.5)	17 (9.3)	1 (0.5)	161 (87.5)	2 (1.1)	2 (1.1)	0.075	4 (2.2)	82 (44.6)	74 (40.2)	24 (13)	0.217
Blood pressure changes	74	32 (43.2)	42 (56.8)	0.223	44 (59.5)	30 (40.5)	**<0.001**	1 (1.3)	6 (8.1)	1 (1.3)	62 (83.8)	3 (4.1)	1 (1.4)	0.095		31 (41.9)	17 (23)	26 (35.1)	**<0.001**
Confusion	61	22 (36.1)	39 (63.9)	0.999	45 (73.8)	16 (26.2)	0.416	--	4 (6.6)	1 (1.6)	53 (86.9)	2 (3.3)	1 (1.6)	0.225	1 (1.6)	27 (44.3)	21 (34.4)	12 (19.7)	0.598
Diarrhea	56	11 (19.6)	45 (80.4)	0.013	47 (83.9)	9 (16.1)	0.418	1 (1.8)	2 (3.6)	1 (1.8)	50 (89.2)	2 (3.6)	--	0.281	2 (3.6)	30 (53.6)	15 (26.8)	9 (16.1)	0.724
Difficulty concentrating	387	113 (29.2)	274 (70.8)	**<0.001**	298 (77.0)	89 (23.0)	0.34	3 (0.8)	13 (3.4)	1 (0.2)	362 (93.5)	5 (1.3)	3 (0.8)	0.078	8 (2.1)	191 (49.4)	125 (32.3)	63 (16.3)	0.468
Speech disturbance	241	77 (31.9)	164 (68.1)	0.159	191 (79.3)	50 (20.7)	0.891	1 (0.4)	9 (3.7)	1 (0.4)	227 (94.3)	1 (0.4)	2 (0.8)	0.292	7 (2.9)	114 (47.3)	79 (32.8)	41 (17)	0.586
Decreased visual acuity	147	42 (28.6)	105 (71.4)	0.054	114 (77.6)	33 (22.4)	0.791	1 (0.7)	7 (4.7)	1 (0.7)	133 (90.5)	3 (2.0)	2 (1.4)	0.368	5 (3.4)	69 (46.9)	43 (29.3)	30 (20.4)	0.119
Loss of muscle strength	170	68 (40.0)	102 (60.0)	0.274	129 (75.9)	41 (24.1)	0.376	1 (0.6)	18 (10.6)	2 (1.2)	145 (85.3)	3 (1.7)	1 (0.6)	**0.032**	5 (2.9)	62 (36.5)	79 (46.5)	24 (14.1)	**0.001**
Decreased libido/sexual desire	115	44 (38.3)	71 (61.7)	0.666	89 (77.4)	26 (22.6)	0.802	1 (0.9)	12 (10.4)	3 (2.6)	95 (82.6)	4 (3.5)	--	**0.001**	2 (1.7)	47 (40.9)	50 (43.5)	16 (13.9)	0.106
Abdominal pain	70	18 (25.7)	52 (74.3)	0.084	54 (77.1)	16 (22.9)	0.854	--	3 (4.3)	1 (1.4)	63 (90.0)	2 (2.9)	1 (1.4)		0.377	35 (50)	27 (38.6)	8 (11.4)	0.625
Headache	354	97 (27.4)	257 (72.6)	**<0.001**	279 (78.8)	75 (21.2)	0.999	4 (1.1)	20 (5.7)	3 (0.8)	318 (89.9)	5 (1.4)	4 (1.1)	**0.033**	8 (2.3)	182 (51.4)	117 (33.1)	47 (13.3)	0.764
Chest pain	187	71 (38.0)	116 (62.0)	0.595	157 (84.0)	30 (16.0)	0.068	1 (0.5)	19 (10.2)	2 (1.1)	162 (86.6)	1 (0.5)	2 (1.1)	0.011	3 (1.6)	95 (50.8)	68 (36.4)	21 (11.2)	0.381
Burning sensation in any part of the body	75	25 (33.3)	50 (66.7)	0.708	55 (73.3)	20 (26.7)	0.301	2 (2.7)	5 (6.7)	1 (1.3)	66 (88.0)	--	1 (1.3)	0.052		28 (37.3)	29 (38.7)	18 (24)	**0.018**
Unusual muscle aches	109	44 (40.4)	65 (59.6)	0.37	80 (73.4)	29 (26.6)	0.191	1 (0.9)	9 (8.3)	2 (1.8)	95 (87.2)	2 (1.8)	--	0.321	1 (0.9)	44 (40.4)	47 (43.1)	17 (15.6)	0.078
Usual muscle aches	164	54 (32.9)	110 (67.1)	0.423	123 (75.0)	41 (25.0)	0.246	--	7 (4.3)	2 (1.2)	151 (92.1)	3 (1.8)	1 (0.6)	0.792	3 (1.8)	66 (40.2)	64 (39)	31 (18.9)	**0.041**
Shaking chills	45	11 (24.4)	34 (75.6)	0.136	37 (82.2)	8 (17.8)	0.689	1 (2.2)	1 (2.2)	--	40 (89.0)	2 (4.4)	1 (2.2)	**0.026**		26 (57.8)	14 (31.1)	5 (11.1)	0.598
Sneezing	92	31 (33.7)	61 (66.3)	0.713	80 (87.0)	12 (13.0)	0.059	1 (1.1)	4 (4.3)	2 (2.2)	83 (90.2)	2 (2.2)	--	0.391	2 (2.2)	50 (54.3)	27 (29.3)	13 (14.1)	0.777
Fatigue or tiredness	652	221 (33.9)	431 (66.1)	0.091	500 (77.0)	152 (23.0)	0.054	4 (0.6)	42 (6.4)	7 (1.1)	585 (89.7)	11 (1.7)	3 (0.5)	0.129	12 (1.8)	325 (49.8)	221 (33.9)	94 (14.4)	0.266
Tingling in extremities	137	50 (36.5)	87 (63.5)	0.972	106 (77.4)	31 (22.6)	0.762	--	16 (11.7)	4 (2.9)	113 (82.5)	2 (1.4)	2 (1.5)	**<0.001**	2 (1.5)	47 (34.3)	63 (46.0)	25 (18.2)	**0.001**
Insomnia	342	107 (31.3)	235 (68.7)	**0.034**	259 (75.7)	83 (24.3)	0.12	3 (0.9)	19 (5.6)	--	312 (91.2)	6 (1.7)	2 (0.6)	0.693	8 (2.3)	170 (49.7)	111 (32.5)	53 (15.5)	0.851
Heat intolerance	20	7 (35.0)	13 (65.0)	0.999	12 (60.0)	8 (40.0)	0.073	--	1 (5.0)	--	19 (95.0)	--	--	0.999		7 (35)	9 (45)	4 (20)	0.361
Cold intolerance	92	28 (30.4)	64 (69.6)	0.294	74 (80.4)	18 (19.6)	0.776	--	3 (3.2)	--	89 (96.8)	--	--	0.448	2 (2.2)	41 (44.6)	34 (37)	15 (16.3)	0.758
Nausea	68	10 (14.7)	58 (85.3)	**<0.001**	52 (76.5)	16 (23.5)	0.752	1 (1.5)	2 (2.9)	1 (1.5)	62 (91.2)	2 (2.9)	--	0.434	1 (1.5)	41 (60.3)	20 (29.4)	6 (8.8)	0.259
Neuritis	96	22 (22.9)	74 (77.1)	**0.007**	65 (67.7)	31 (32.3)	**0.008**	2 (2.1)	5 (5.2)	1 (1.0)	87 (90.7)	1 (1.0)	--	0.31	2 (2.1)	37 (38.5)	38 (39.6)	19 (19.8)	0.113

Palpitations	182	59 (32.4)	123 (67.6)	0.308	139 (76.4)	43 (23.6)	0.453	1 (0.6)	8 (4.4)	3 (1.7)	163 (89.4)	4 (2.2)	3 (1.7)	**0.015**	2 (1.1)	83 (45.6)	69 (37.9)	28 (15.4)	0.261
Facial paralysis	7	4 (57.1)	3 (42.9)	0.438	6 (85.7)	1 (14.3)	0.999	--	--	1 (14.3)	6 (85.7)	--	--	0.056		3 (42.9)		4 (57.1)	0.08
Loss of appetite	55	13 (23.6)	42 (76.4)	0.069	46 (83.6)	9 (16.4)	0.457	--	2 (3.6)	1 (1.8)	49 (89.1)	3 (5.5)	--	**0.039**		32 (58.2)	20 (36.4)	3 (5.5)	0.129
Hearing loss	49	16 (32.6)	33 (67.4)	0.728	35 (71.4)	14 (28.6)	0.272	--	2 (4.1)	1 (2.0)	45 (91.9)	1 (2.0)	--	0.675	1 (2)	18 (36.7)	20 (40.8)	10 (20.4)	0.277
Hair loss	391	75 (19.2)	316 (80.8)	**<0.001**	303 (77.5)	88 (22.5)	0.506	2 (0.5)	23 (5.9)	2 (0.5)	353 (90.2)	10 (2.6)	1 (0.3)	0.195	16 (4.1)	198 (50.6)	123 (31.5)	54 (13.8)	0.113
Loss of taste	172	59 (34.3)	113 (65.7)	0.675	135 (78.5)	37 (21.5)	0.999	1 (0.6)	9 (5.2)	--	155 (90.1)	6 (3.5)	1 (0.6)	0.134	9 (5.2)	89 (51.7)	54 (31.4)	20 (11.6)	0.079
Memory deficits	287	84 (29.3)	203 (70.7)	**0.007**	229 (79.8)	58 (20.2)	0.668	2 (0.7)	13 (4.5)	1 (0.4)	267 (93.0)	2 (0.7)	2 (0.7)	0.554	10 (3.5)	130 (45.3)	101 (35.2)	46 (16)	0.323
Muscle loss	97	48 (49.5)	49 (50.5)	**0.005**	77 (79.4)	20 (20.6)	0.972	2 (2.1)	7 (7.3)	1 (1.0)	86 (88.6)	1 (1.0)	--	0.242	2 (2.1)	44 (45.4)	34 (35.1)	17 (17.5)	0.728
Loss of smell	263	90 (34.2)	173 (65.8)	0.538	203 (77.2)	60 (22.8)	0.539	1 (0.4)	11 (4.2)	2 (0.8)	243 (92.3)	5 (1.9)	1 (0.4)	0.871	9 (3.4)	144 (54.8)	80 (30.4)	30 (11.4)	0.117
Body hair loss	14	7 (50.0)	7 (50.0)	0.413	8 (57.1)	6 (42.9)	0.097	--	--	--	14 (100.0)	--	--	**<0.001**		10 (71.4)	2 (14.3)	2 (14.3)	0.238
Pruritus (itching)	114	40 (35.1)	74 (64.9)	0.911	88 (77.2)	26 (22.8)	0.763	2 (1.8)	10 (8.8)	3 (2.6)	96 (84.1)	2 (1.8)	1 (0.9)	**0.013**	2 (1.8)	61 (53.5)	33 (28.9)	18 (15.8)	0.619
Gastroesophageal reflux	87	35 (40.2)	52 (59.8)	0.459	65 (74.7)	22 (25.3)	0.413	1 (1.2)	12 (13.8)	1 (1.2)	73 (83.8)	--	--	**0.003**		30 (34.5)	41 (47.1)	16 (18.4)	**0.006**
Skin dryness	139	22 (15.8)	117 (84.2)	**<0.001**	100 (71.9)	39 (28.1)	**0.047**	1 (0.7)	6 (4.3)	--	128 (92.1)	4 (2.9)	--	0.384	1 (0.7)	73 (52.5)	45 (32.4)	20 (14.4)	0.46
Thrist	77	32 (41.6)	45 (58.4)	0.352	65 (84.4)	12 (15.6)	0.263	1 (1.3)	12 (15.6)	1 (1.3)	60 (77.9)	2 (2.6)	1 (1.3)	**<0.001**	1 (1.3)	37 (48.1)	30 (39)	9 (11.7)	0.629
Easy crying	202	43 (21.3)	159 (78.7)	**<0.001**	159 (78.7)	43 (21.3)	0.999	2 (1.0)	7 (3.5)	2 (1.0)	186 (92.0)	4 (2.0)	1 (0.5)	0.607	4 (2)	95 (47)	76 (37.6)	27 (13.4)	0.552
Excessive sweating	90	30 (33.3)	60 (66.7)	0.663	59 (65.6)	31 (34.4)	**0.002**	--	--	--	87 (96.7)	2 (2.2)	1 (1.1)	0.42		36 (40)	35 (38.9)	19 (21.1)	0.063
Tremor of the extremities	90	23 (25.6)	67 (74.4)	**0.041**	63 (70.0)	27 (30.0)	**0.048**	2 (2.2)	2 (2.2)	3 (3.3)	81 (90.1)	1 (1.1)	1 (1.1)	**0.003**	1 (1.1)	46 (51.1)	36 (40)	7 (7.8)	0.164
Persistent cough	245	85 (34.7)	160 (65.3)	0.683	196 (80.0)	49 (20.0)	0.643	2 (0.8)	22 (9.0)	2 (0.8)	218 (89.0)	1 (0.4)	--	**0.033**	5 (2)	127 (51.8)	86 (35.1)	27 (11)	0.325
Brittle nails	113	8 (7.1)	105 (92.9)	**<0.001**	94 (83.2)	19 (16.8)	0.271	--	4 (3.5)	--	106 (93.8)	3 (2.7)	--	0.313	2 (1.8)	64 (56.6)	33 (29.2)	14 (12.4)	0.436
Vomit	25	6 (24.0)	19 (76.0)	0.292	18 (72.0)	7 (28.0)	0.559	--	--	1 (4.0)	23 (92)	1 (4.0)	--	0.097		18 (72)	5 (20)	2 (8)	0.099
**Total**	**7746**	**2341**	**5405**		**5974**	**1772**		**56**	**448**	**74**	**6984**	**136**	**48**		**178**	**3760**	**2669**	**1139**	

Supplementary file S3. Distribution of self-reported long-COVID symptoms by age group among 1100 patients.

Symptom	Total n	0 to 10 n (%)	10 to 20 n (%)	20 to 30 n (%)	30 to 40 n (%)	40 to 50 n (%)	50 to 60 n (%)	60 to 70 n (%)	70 to 80 n (%)	80 to 90 n (%)	p Value
Alopecia	32	--	--	13 (40.6)	11 (34.4)	5 (15.6)	3 (9.4)	--	--	--	0.908
Taste dysfunction	199	--	22 (11.1)	84 (42.2)	52 (26.1)	30 (15.1)	8 (4.0)	1 (0.5)	2 (1.0)	--	**<0.001**
Alterations in glucose	16	--	1 (6.3)	3 (18.8)	4 (25.0)	5 (31.3)	2 (12.5)	--	1 (6.3)	--	**0.017**
Menstrual cycle alterations	118	--	6 (5.1)	40 (33.9)	47 (39.8)	23 (19.5)	2 (1.7)	--	--	--	0.172
Hallucinations	20	--	--	6 (30.0)	11 (55.0)	1 (5.0)	1 (5.0)	1 (5.0)	--	--	0.185
Anxiety	379	--	27 (7.1)	141 (37.2)	119 (31.4)	65 (17.2)	22 (5.8)	3 (0.8)	2 (0.5)	--	0.334
Brittle hair	111	--	8 (7.2)	44 (39.6)	40 (36.0)	10 (9.0)	8 (7.2)	1 (0.9)	--	--	0.227
Mood changes	234	1 (0.43)	15 (6.4)	92 (39.3)	76 (32.5)	39 (16.7)	9 (3.9)	2 (0.9)	--	--	0.122
Tachycardia	184	--	16 (8.7)	54 (29.4)	70 (38.0)	28 (15.2)	11 (6.0)	4 (2.2)	1 (0.5)	--	0.173
Blood pressure changes	74	--	5 (6.8)	20 (27.0)	17 (23.0)	21 (28.4)	7 (9.5)	4 (5.4)	--	--	**0.001**
Confusion	61	--	3 (4.9)	15 (24.6)	28 (45.9)	10 (16.4)	2 (3.3)	3 (4.9)	--	--	**0.039**
Diarrhea	56	--	4 (7.1)	12 (21.4)	26 (46.4)	11 (19.6)	3 (5.4)	--	--	--	0.182
Difficulty concentrating	387	--	28 (7.2)	152 (39.3)	131 (33.9)	53 (13.7)	21 (5.4)	2 (0.5)	--	--	**0.007**
Speech disturbance	241	--	8 (3.3)	78 (32.4)	98 (40.7)	38 (15.8)	16 (6.6)	3 (1.2)	--	--	0.169
Decreased visual acuity	147	--	6 (4.1)	36 (24.5)	47 (32.0)	39 (26.5)	14 (9.5)	5 (3.4)	--	--	**<0.001**
Loss of muscle strength	170	--	7 (4.1)	51 (30.0)	65 (38.2)	23 (13.5)	16 (9.4)	5 (2.9)	3 (1.8)	--	**0.011**
Decreased libido/sexual desire	115	--	1 (0.9)	33 (28.7)	45 (39.1)	27 (23.5)	8 (7.0)	1 (0.9)	--	--	0.067
Abdominal pain	70	--	6 (8.6)	27 (38.6)	21 (30.0)	7 (10.0)	6 (8.6)	2 (2.9)	1 (1.4)	--	0.316
Headache	354	--	23 (6.5)	134 (37.9)	119 (33.6)	55 (15.5)	19 (5.4)	4 (1.1)	--	--	0.442
Chest pain	187	--	15 (8.0)	59 (31.6)	73 (39.0)	22 (11.8)	15 (8.0)	2 (1.1)	--	1 (0.5)	**0.021**
Burning sensation in any part of the body	75	--	4 (5.3)	21 (28.0)	23 (30.7)	15 (20.0)	9 (12.0)	3 (4.0)	--	--	0.098
Unusual muscle aches	109	--	6 (5.5)	26 (23.9)	36 (33.0)	24 (22.0)	13 (11.9)	3 (2.8)	1 (0.9)	--	**0.043**
Usual muscle aches	164	--	12 (7.3)	40 (24.4)	65 (39.6)	26 (15.9)	16 (9.8)	4 (2.4)	1 (0.6)	--	**0.029**
Shaking chills	45	--	10 (22.2)	14 (31.1)	13 (28.9)	4 (8.9)	4 (8.9)	--	--	--	**<0.001**
Sneezing	92	--	10 (10.9)	33 (35.9)	30 (32.6)	11 (12.0)	6 (6.5)	1 (1.09)	--	1 (1.1)	**0.006**
Fatigue or tiredness	652	1 (0.15)	35 (5.4)	237 (36.4)	209 (32.1)	112 (17.2)	40 (6.1)	11 (1.7)	6 (0.9)	1 (0.2)	0.274
Tingling in extremities	137	--	8 (5.8)	29 (21.2)	58 (42.3)	21 (15.3)	16 (11.7)	3 (2.2)	2 (1.5)	--	**0.001**
Insomnia	342	--	24 (7.0)	113 (33.0)	112 (32.8)	59 (17.3)	28 (8.2)	4 (1.2)	1 (0.3)	1 (0.3)	0.353
Heat intolerance	20	--	--	9 (45.0)	6 (30.0)	--	4 (20.0)	1 (5.0)	--	--	0.154
Cold intolerance	92	--	6 (6.5)	30 (32.6)	28 (30.4)	18 (19.6)	8 (8.7)	2 (2.17)	--	--	0.831
Nausea	68	--	9 (13.2)	31 (45.6)	19 (27.9)	4 (5.9)	3 (4.4)	--	2 (2.9)	--	**<0.001**
Neuritis	96	--	1 (1.0)	18 (18.8)	33 (34.4)	26 (27.1)	15 (15.6)	2 (2.1)	1 (1.0)	--	**<0.001**
Palpitations	182	--	8 (4.4)	55 (30.2)	70 (38.5)	31 (17.0)	14 (7.7)	3 (1.7)	1 (0.6)	--	0.719
Facial paralysis	7	--	--	2 (28.6)	2 (28.6)	2 (28.6)	--	1 (14.3)	--	--	0.046
Loss of appetite	55	--	11 (20.0)	18 (32.7)	17 (30.9)	5 (9.1)	2 (3.6)	--	1 (1.8)	1 (1.8)	**<0.001**
Hearing loss	49	--	2 (4.1)	13 (26.5)	15 (30.6)	10 (20.4)	8 (16.3)	1 (2.0)	--	--	0.101
Hair loss	391	--	25 (6.4)	140 (35.8)	129 (33.0)	68 (17.4)	24 (6.1)	5 (1.3)	--	--	0.918
Loss of taste	172	--	22 (12.8)	60 (34.9)	46 (26.7)	27 (15.7)	12 (7.0)	3 (1.7)	2 (1.2)	--	**<0.001**
Memory deficits	287	--	10 (3.5)	90 (31.4)	121 (42.2)	43 (15.0)	21 (7.3)	2 (0.7)	--	--	**0.011**
Muscle loss	97	--	6 (6.2)	33 (34.0)	33 (34.0)	15 (15.5)	6 (6.2)	3 (3.1)	1 (1.0)	--	0.789
Loss of smell	263	--	28 (10.7)	98 (37.3)	75 (28.5)	40 (15.2)	17 (6.5)	5 (1.9)	--	--	**<0.001**
Body hair loss	14	--	--	5 (35.7)	6 (42.9)	2 (14.3)	1 (7.1)	--	--	--	0.957
Pruritus (itching)	114	--	6 (5.3)	26 (22.8)	42 (36.8)	28 (24.6)	8 (7.0)	4 (3.5)	--	--	**0.018**
Gastroesophageal reflux	87	--	2 (2.3)	24 (27.6)	36 (41.4)	16 (18.4)	7 (8.1)	2 (2.3)	--	--	0.341

Skin dryness	139	--	10 (7.2)	38 (27.3)	55 (39.6)	20 (14.4)	13 (9.4)	2 (1.4)	1 (0.7)	--	0.257
Thrist	77	--	12 (15.6)	24 (31.2)	34 (44.2)	6 (7.8)	1 (1.3)	--	--	--	**<0.001**
Easy crying	202	1 (0.5)	19 (9.4)	73 (36.1)	70 (34.7)	27 (13.4)	11 (5.5)	1 (0.5)	--	--	**0.015**
Excessive sweating	90	--	7 (7.8)	16 (17.8)	32 (35.6)	26 (28.9)	7 (7.8)	2 (2.2)	--	--	**0.004**
Tremor of the extremities	90	--	9 (10.0)	30 (33.3)	31 (34.4)	10 (11.1)	9 (10.0)	1 (1.1)	--	--	0.169
Persistent cough	245	1 (0.41)	13 (5.3)	94 (38.4)	89 (36.3)	34 (13.9)	11 (4.5)	--	2 (0.8)	1 (0.4)	0.066
Brittle nails	113	--	13 (11.5)	35 (31.0)	32 (28.3)	20 (17.7)	10 (8.9)	2 (1.8)	1 (0.9)	--	0.074
Vomit	25	--	5 (20.0)	12 (48.0)	8 (32.0)	--	--	--	--	--	**0.036**
**Total**	**7746**	**4**	**534**	**2581**	**2675**	**1262**	**537**	**114**	**33**	**6**	

(A)

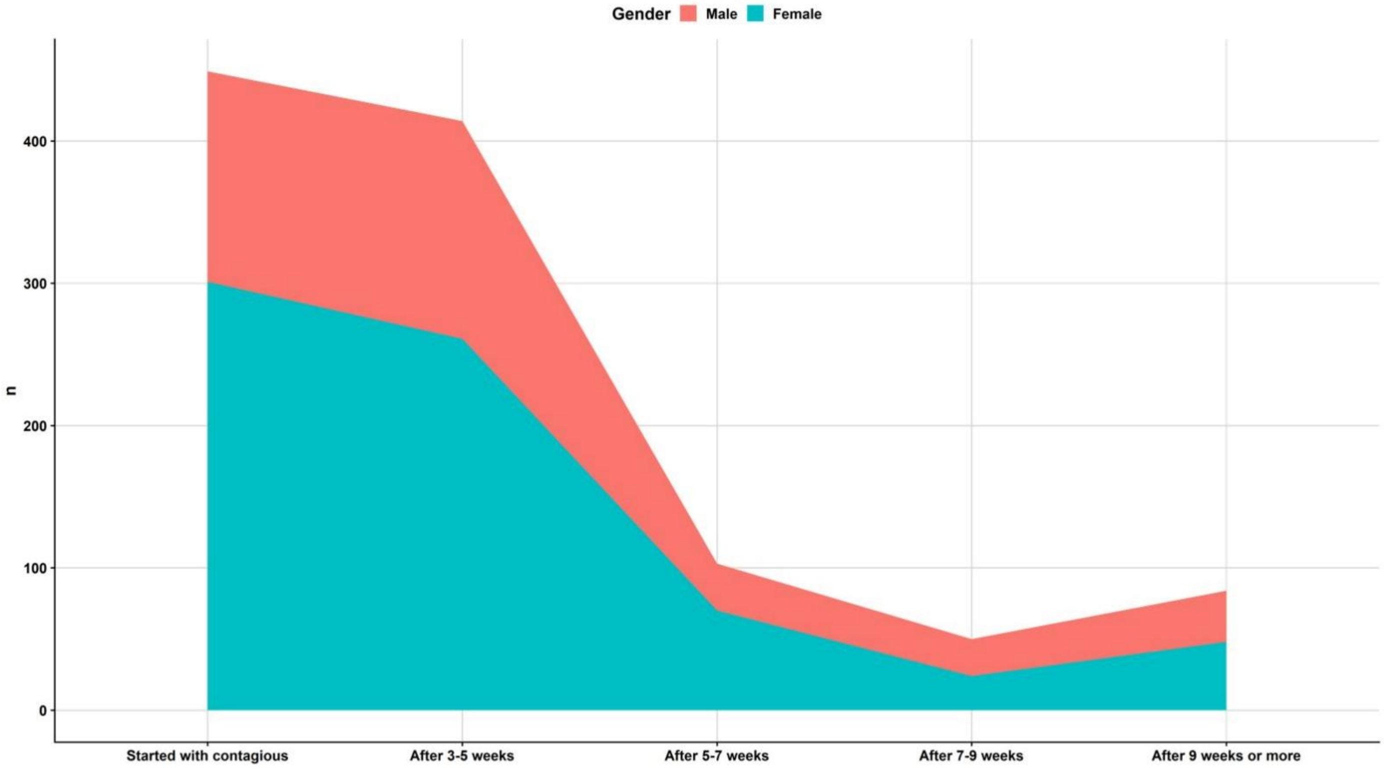

(B)

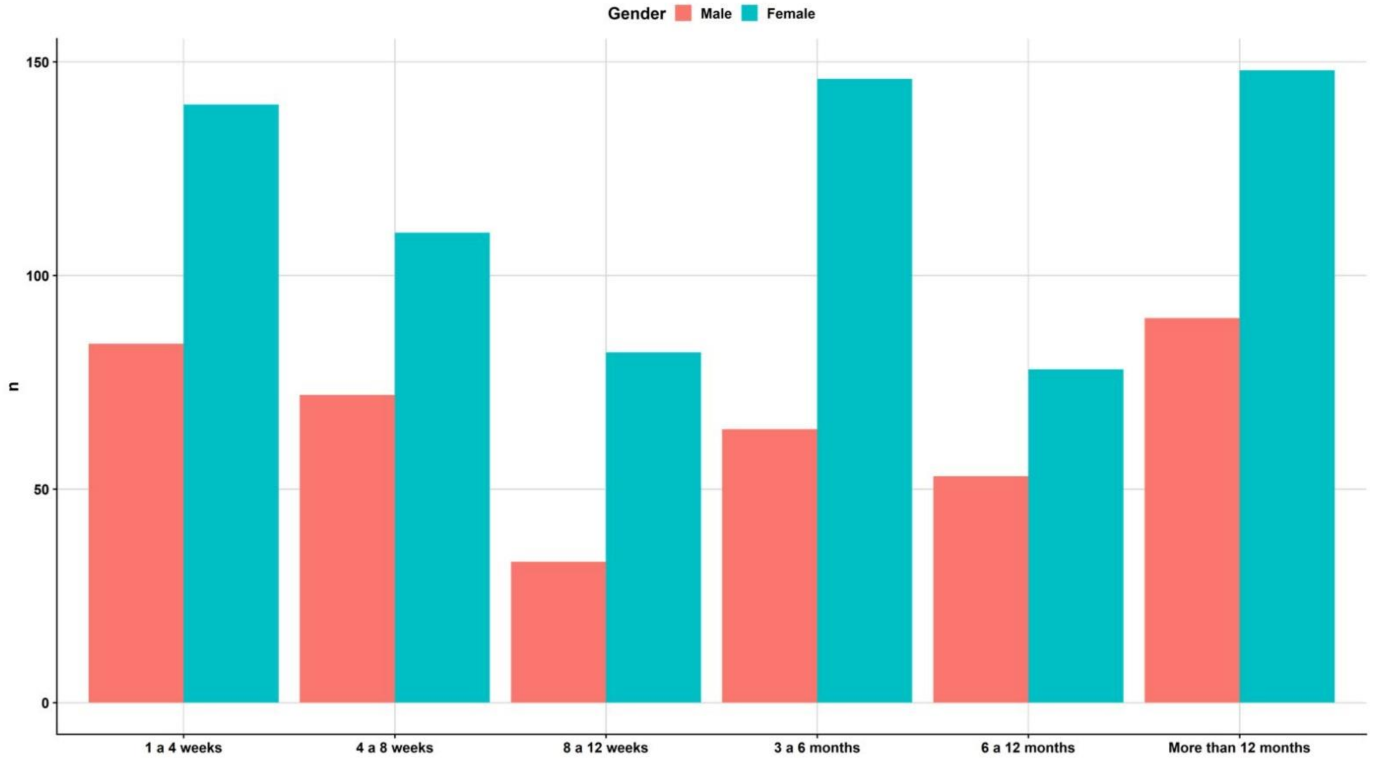

Supplementary file S4. Behavior of long-COVID symptoms according to sex. **(A).** Shows the frequency regarding the time of onset of self-referred symptoms from the onset of infection until after 9 weeks of infection. **(B).** Shows the frequency regarding the duration of symptoms.

Supplementary file S5. Distribution of COVID-19 post-acute symptom characteristics according to the characteristics of Ecuadorian participants.

**Symptoms**	**Onset time n (%)**						**Symptoms duration n (%)**							**Frequency of presentation n (%)**							**Symptoms evolution n (%)**				
	**Initiated with infection**	**3 to 5 weeks after infection**	**5 to 7 weeks after infection**	**7 to 9 weeks after infection**	**> 9 weeks of infection**	**p Value**	**Between 1 to 4 weeks**	**Between 4 to 8 weeks**	**Between 8 to 12 weeks**	**Between 3 to 6 months**	**Between 6 to 12 months**	**> 12 months**	**p Value**	**Daily**	**> 3 days a week**	**Once a week**	**Once every two weeks**	**Once a month**	**No apply**	**p Value**	**Intermittent (appear - disappear)**	**Gotten worse**	**Improved**	**Has been consistent**	**p Value**
Alopecia	8 (25)	15 (46.9)	1 (3.1)	4 (12.5)	4 (12.5)	**0.044**	2 (6.2)	4 (12.5)	4 (12.5)	7 (21.9)	5 (15.6)	10 (31.2)	0.346	9 (28.1)	13 (40.6)	5 (15.6)	--	4 (12.5)	1 (3.1)	0.512	9 (28.1)	5 (15.6)	9 (28.1)	9 (28.1)	**0.023**
Taste dysfunction	98 (49.2)	57 (28.6)	19 (9.5)	10 (5)	15 (7.5)	**0.048**	23 (11.6)	29 (14.6)	14 (7.0)	36 (18.1)	35 (17.6)	62 (31.2)	**<0.001**	71 (35.7)	42 (21.1)	40 (20.1)	19 (9.5)	14 (7)	13 (6.5)	0.171	52 (26.1)	6 (3)	88 (44.2)	53 (26.6)	**0.03**
Alterations in glucose	5 (31.2)	5 (31.2)	4 (25)	--	2 (12.5)	0.171	2 (12.5)	4 (25)	1 (6.2)	4 (25)	2 (12.5)	3 (18.8)	0.871	6 (37.5)	4 (25)	4 (25)	--	--	2 (12.5)	0.88	2 (12.5)	1 (6.2)	10 (62.5)	3 (18.8)	0.121
Menstrual cycle alterations	51 (43.2)	46 (39)	12 (10.2)	3 (2.5)	6 (5.1)	0.62	17 (14.4)	14 (11.9)	18 (15.3)	34 (28.8)	12 (10.2)	23 (19.5)	**0.014**	30 (25.4)	33 (28)	30 (25.4)	10 (8.5)	10 (8.5)	5 (4.2)	0.455	38 (32.2)	3 (2.5)	38 (32.2)	39 (33.1)	0.528
Hallucinations	8 (40)	6 (30)	2 (10)	2 (10)	2 (10)	0.77	2 (10)	5 (25)	3 (15)	2 (10)	3 (15)	5 (25)	0.622	5 (25)	6 (30)	5 (25)	2 (10)	--	2 (10)	0.945	8 (40)	3 (15)	4 (20)	5 (25)	0.059
Anxiety	146 (38.5)	150 (39.6)	46 (12.1)	13 (3.4)	24 (6.3)	0.062	69 (18.2)	53 (14)	49 (12.9)	74 (19.5)	46 (12.1)	88 (23.2)	0.17	94 (24.8)	106 (28)	99 (26.1)	27 (7.1)	32 (8.4)	21 (5.5)	**0.001**	116 (30.6)	20 (5.3)	114 (30.1)	129 (34)	0.063
Brittle hair	42 (37.8)	44 (39.6)	14 (12.6)	4 (3.6)	7 (6.3)	0.677	10 (9)	15 (13.5)	20 (18)	27 (24.3)	16 (14.4)	23 (20.7)	**0.003**	32 (28.8)	32 (28.8)	25 (22.5)	10 (9)	11 (9.9)	1 (0.9)	0.182	38 (34.2)	8 (7.2)	27 (24.3)	38 (34.2)	0.05
Mood changes	92 (39.3)	94 (40.2)	30 (12.8)	6 (2.6)	12 (5.1)	0.05	52 (22.2)	38 (16.2)	25 (10.7)	35 (15)	26 (11.1)	58 (24.8)	0.451	58 (24.8)	70 (29.9)	61 (26.1)	17 (7.3)	17 (7.3)	11 (4.7)	**0.025**	76 (32.5)	16 (6.8)	65 (27.8)	77 (32.9)	**0.019**
Tachycardia	72 (39.1)	80 (43.5)	18 (9.8)	7 (3.8)	7 (3.8)	0.151	27 (14.7)	30 (16.3)	14 (7.6)	40 (21.7)	25 (13.6)	48 (26.1)	0.126	42 (22.8)	57 (31)	53 (28.8)	11 (6)	15 (8.2)	6 (3.3)	**0.001**	67 (36.4)	10 (5.4)	53 (28.8)	54 (29.3)	**0.048**
Blood pressure changes	27 (36.5)	29 (39.2)	6 (8.1)	7 (9.5)	5 (6.8)	0.303	10 (13.5)	13 (17.6)	9 (12.2)	15 (20.3)	8 (10.8)	19 (25.7)	0.725	19 (25.7)	24 (32.4)	17 (23)	5 (6.8)	5 (6.8)	4 (5.4)	0.773	23 (31.1)	3 (4.1)	24 (32.4)	24 (32.4)	0.936
Confusion	28 (45.9)	19 (31.1)	9 (14.8)	1 (1.6)	4 (6.6)	0.35	10 (16.4)	7 (11.5)	6 (9.8)	9 (14.8)	5 (8.2)	24 (39.3)	**0.031**	16 (26.2)	19 (31.1)	17 (27.9)	5 (8.2)	4 (6.6)	--	0.584	16 (26.2)	6 (9.8)	20 (32.8)	19 (31.1)	0.236
Diarrhea	32 (57.1)	14 (25)	8 (14.3)	1 (1.8)	1 (1.8)	**0.021**	13 (23.2)	4 (7.1)	4 (7.1)	10 (17.9)	13 (23.2)	12 (21.4)	0.064	15 (26.8)	12 (21.4)	12 (21.4)	3 (5.4)	11 (19.6)	3 (5.4)	0.093	19 (33.9)	5 (8.9)	18 (32.1)	14 (25)	0.263
Difficulty concentrating	161 (41.6)	145 (37.5)	45 (11.6)	11 (2.8)	25 (6.5)	0.082	61 (15.8)	51 (13.2)	43 (11.1)	82 (21.2)	48 (12.4)	102 (26.4)	**0.002**	108 (27.9)	116 (30)	80 (20.7)	37 (9.6)	31 (8)	15 (3.9)	**0.014**	118 (30.5)	22 (5.7)	129 (33.3)	118 (30.5)	0.362
Speech disturbance	98 (40.7)	90 (37.3)	28 (11.6)	7 (2.9)	18 (7.5)	0.474	29 (12)	31 (12.9)	30 (12.4)	53 (22)	34 (14.1)	64 (26.6)	**0.001**	62 (25.7)	83 (34.4)	48 (19.9)	26 (10.8)	11 (4.6)	11 (4.6)	**<0.001**	81 (33.6)	14 (5.8)	74 (30.7)	72 (29.9)	0.124
Decreased visual acuity	53 (36.1)	56 (38.1)	22 (15)	6 (4.1)	10 (6.8)	0.142	13 (8.8)	13 (8.8)	12 (8.2)	30 (20.4)	24 (16.3)	55 (37.4)	**<0.001**	42 (28.6)	41 (27.9)	40 (27.2)	9 (6.1)	9 (6.1)	6 (4.1)	0.112	52 (35.4)	10 (6.8)	37 (25.2)	48 (32.7)	**0.022**
Loss of muscle strength	69 (40.6)	72 (42.4)	16 (9.4)	5 (2.9)	8 (4.7)	0.331	20 (11.8)	36 (21.2)	21 (12.4)	31 (18.2)	19 (11.2)	43 (25.3)	**0.034**	35 (20.6)	50 (29.4)	47 (27.6)	15 (8.8)	14 (8.2)	9 (5.3)	**0.015**	67 (39.4)	8 (4.7)	48 (28.2)	47 (27.6)	**0.008**
Decreased libido/sexual desire	38 (33)	55 (47.8)	9 (7.8)	6 (5.2)	7 (6.1)	0.177	16 (13.9)	19 (16.5)	16 (13.9)	20 (17.4)	16 (13.9)	28 (24.3)	0.396	26 (22.6)	34 (29.6)	35 (30.4)	8 (7)	10 (8.7)	2 (1.7)	0.009	46 (40)	4 (3.5)	31 (27)	34 (29.6)	**0.033**
Abdominal pain	30 (42.9)	24 (34.3)	6 (8.6)	3 (4.3)	7 (10)	0.923	16 (22.9)	7 (10)	11 (15.7)	13 (18.6)	6 (8.6)	17 (24.3)	0.404	12 (17.1)	22 (31.4)	22 (31.4)	3 (4.3)	6 (8.6)	5 (7.1)	0.049	28 (40)	2 (2.9)	23 (32.9)	17 (24.3)	0.164
Headache	161 (45.5)	118 (33.3)	35 (9.9)	15 (4.2)	25 (7.1)	0.211	79 (22.3)	61 (17.2)	36 (10.2)	68 (19.2)	37 (10.5)	73 (20.6)	0.798	74 (20.9)	107 (30.2)	85 (24)	29 (8.2)	39 (11)	20 (5.6)	**<0.001**	106 (29.9)	22 (6.2)	118 (33.3)	108 (30.5)	0.24
Chest pain	75 (40.1)	82 (43.9)	15 (8)	6 (3.2)	9 (4.8)	0.198	30 (16)	43 (23)	22 (11.8)	41 (21.9)	19 (10.2)	32 (17.1)	**0.038**	41 (21.9)	51 (27.3)	56 (29.9)	12 (6.4)	17 (9.1)	10 (5.3)	0.002	66 (35.3)	14 (7.5)	53 (28.3)	54 (28.9)	**0.01**
Burning sensation in any part of the body	33 (44)	29 (38.7)	9 (12)	1 (1.3)	3 (4)	0.401	13 (17.3)	6 (8)	9 (12)	18 (24)	8 (10.7)	21 (28.0)	0.241	16 (21.3)	26 (34.7)	19 (25.3)	7 (9.3)	5 (6.7)	2 (2.7)	0.162	24 (32)	6 (8)	21 (28)	24 (32)	0.301

Unusual muscle aches	48 (44)	38 (34.9)	11 (10.1)	4 (3.7)	8 (7.3)	0.932	20 (18.3)	11 (10.1)	12 (11)	21 (19.3)	16 (14.7)	29 (26.6)	0.359	26 (23.9)	33 (30.3)	25 (22.9)	14 (12.8)	7 (6.4)	4 (3.7)	0.088	43 (39.4)	5 (4.6)	28 (25.7)	33 (30.3)	**0.044**
Usual muscle aches	59 (36)	60 (36.6)	20 (12.2)	10 (6.1)	15 (9.1)	0.353	33 (20.1)	28 (17.1)	17 (10.4)	29 (17.7)	23 (14)	34 (20.7)	0.959	33 (20.1)	58 (35.4)	35 (21.3)	18 (11)	12 (7.3)	8 (4.9)	0.006	50 (30.5)	13 (7.9)	54 (32.9)	47 (28.7)	0.12
Shaking chills	27 (60)	14 (31.1)	4 (8.9)	--	--	0.172	12 (26.7)	4 (8.9)	10 (22.2)	4 (8.9)	5 (11.1)	10 (22.2)	**0.044**	9 (20)	11 (24.4)	10 (22.2)	4 (8.9)	8 (17.8)	3 (6.7)	0.284	17 (37.8)	2 (4.4)	15 (33.3)	11 (24.4)	0.549
Sneezing	47 (51.1)	34 (37)	9 (9.8)	--	2 (2.2)	0.108	24 (26.1)	15 (16.3)	11 (12)	10 (10.9)	14 (15.2)	18 (19.6)	0.259	17 (18.5)	20 (21.7)	31 (33.7)	6 (6.5)	9 (9.8)	9 (9.8)	0.004	33 (35.9)	4 (4.3)	26 (28.3)	29 (31.5)	0.358
Fatigue or tiredness	271 (41.6)	250 (38.3)	69 (10.6)	21 (3.2)	41 (6.3)	**0.011**	143 (21.9)	112 (17.2)	75 (11.5)	130 (19.9)	70 (10.7)	122 (18.7)	0.025	174 (26.7)	192 (29.4)	130 (19.9)	55 (8.4)	63 (9.7)	38 (5.8)	0.011	193 (29.6)	34 (5.2)	225 (34.5)	200 (30.7)	0.461
Tingling in extremities	45 (32.8)	65 (47.4)	13 (9.5)	8 (5.8)	6 (4.4)	0.056	16 (11.7)	19 (13.9)	13 (9.5)	26 (19)	23 (16.8)	40 (29.2)	**0.016**	30 (21.9)	39 (28.5)	42 (30.7)	16 (11.7)	4 (2.9)	6 (4.4)	**<0.001**	48 (35)	7 (5.1)	38 (27.7)	44 (32.1)	0.181
Insomnia	136 (39.8)	139 (40.6)	29 (8.5)	12 (3.5)	26 (7.6)	0.572	62 (18.1)	46 (13.5)	35 (10.2)	68 (19.9)	46 (13.5)	85 (24.9)	0.177	104 (30.4)	108 (31.6)	66 (19.3)	22 (6.4)	27 (7.9)	15 (4.4)	0.086	91 (26.6)	22 (6.4)	111 (32.5)	118 (34.5)	**0.059**
Heat intolerance	8 (40)	7 (35)	3 (15)	1 (5)	1 (5)	0.92	2 (10)	1 (5)	3 (15)	4 (20)	1 (5)	9 (45.0)	0.105	4 (20)	8 (40)	5 (25)	2 (10)	1 (5)	--	0.65	9 (45)	1 (5)	4 (20)	6 (30)	0.353
Cold intolerance	38 (41.3)	30 (32.6)	13 (14.1)	1 (1.1)	10 (10.9)	0.129	14 (15.2)	9 (9.8)	4 (4.3)	22 (23.9)	16 (17.4)	27 (29.3)	**0.0145**	20 (21.7)	35 (38)	21 (22.8)	10 (10.9)	4 (4.3)	2 (2.2)	0.014	27 (29.3)	7 (7.6)	31 (33.7)	27 (29.3)	0.513
Nausea	34 (50)	25 (36.8)	6 (8.8)	--	3 (4.4)	0.519	12 (17.6)	8 (11.8)	10 (14.7)	10 (14.7)	11 (16.2)	17 (25.0)	0.449	18 (26.5)	15 (22.1)	20 (29.4)	4 (5.9)	9 (13.2)	2 (2.9)	0.156	21 (30.9)	2 (2.9)	26 (38.2)	19 (27.9)	0.809
Neuritis	36 (37.5)	39 (40.6)	13 (13.5)	3 (3.1)	5 (5.2)	0.437	12 (12.5)	18 (18.8)	8 (8.3)	20 (20.8)	10 (10.4)	28 (29.2)	0.215	27 (28.1)	32 (33.3)	19 (19.8)	9 (9.4)	6 (6.2)	3 (3.1)	0.415	27 (28.1)	7 (7.3)	30 (31.2)	32 (33.3)	0.495
Palpitations	71 (39)	72 (39.6)	15 (8.2)	7 (3.8)	17 (9.3)	0.782	29 (15.9)	32 (17.6)	21 (11.5)	36 (19.8)	24 (13.2)	40 (22)	0.722	45 (24.7)	55 (30.2)	43 (23.6)	14 (7.7)	15 (8.2)	10 (5.5)	0.376	64 (35.2)	14 (7.7)	57 (31.3)	47 (25.8)	**0.011**
Facial paralysis	3 (42.9)	3 (42.9)	--	--	1 (14.3)	0.875	2 (28.6)	1 (14.3)	--	--	--	4 (57.1)	0.509	3 (42.9)	1 (14.3)	2 (28.6)	--	--	1 (14.3)	0.752	2 (28.6)	--	3 (42.9)	2 (28.6)	0.946
Loss of appetite	32 (58.2)	19 (34.5)	2 (3.6)	1 (1.8)	1 (1.8)	**0.041**	11 (20)	6 (10.9)	9 (16.4)	12 (21.8)	5 (9.1)	12 (21.8)	0.599	16 (29.1)	15 (27.3)	11 (20)	3 (5.5)	4 (7.3)	6 (10.9)	0.854	17 (30.9)	3 (5.5)	17 (30.9)	18 (32.7)	0.905
Hearing loss	18 (36.7)	21 (42.9)	7 (14.3)	--	3 (6.1)	0.601	4 (8.2)	3 (6.1)	7 (14.3)	13 (26.5)	5 (10.2)	17 (34.7)	**0.019**	15 (30.6)	13 (26.5)	13 (26.5)	6 (12.2)	1 (2)	1 (2)	0.215	15 (30.6)	2 (4.1)	14 (28.6)	18 (36.7)	0.746
Hair loss	146 (37.3)	154 (39.4)	40 (10.2)	19 (4.9)	32 (8.2)	0.533	53 (13.6)	52 (13.3)	48 (12.3)	83 (21.2)	54 (13.8)	101 (25.8)	**<0.001**	107 (27.4)	108 (27.6)	87 (22.3)	31 (7.9)	34 (8.7)	24 (6.1)	0.523	115 (29.4)	14 (3.6)	137 (35)	125 (32)	0.698
Loss of taste	92 (53.5)	49 (28.5)	18 (10.5)	4 (2.3)	9 (5.2)	**0.002**	24 (14)	22 (12.8)	20 (11.6)	21 (12.2)	28 (16.3)	57 (33.1)	**<0.001**	54 (31.4)	40 (23.3)	35 (20.3)	11 (6.4)	16 (9.3)	16 (9.3)	0.617	50 (29.1)	8 (4.7)	66 (38.4)	48 (27.9)	0.726
Memory deficits	113 (39.4)	103 (35.9)	33 (11.5)	13 (4.5)	25 (8.7)	0.559	30 (10.5)	40 (13.9)	31 (10.8)	59 (20.6)	45 (15.7)	82 (28.6)	**<0.001**	70 (24.4)	102 (35.5)	60 (20.9)	27 (9.4)	16 (5.6)	12 (4.2)	**<0.001**	91 (31.7)	14 (4.9)	96 (33.4)	86 (30)	0.585
Muscle loss	47 (48.5)	35 (36.1)	10 (10.3)	2 (2.1)	3 (3.1)	0.198	10 (10.3)	10 (10.3)	10 (10.3)	20 (20.6)	16 (16.5)	31 (32.0)	0.01	24 (24.7)	35 (36.1)	22 (22.7)	8 (8.2)	2 (2.1)	6 (6.2)	0.065	31 (32)	7 (7.2)	37 (38.1)	22 (22.7)	0.183
Loss of smell	135 (51.3)	76 (28.9)	27 (10.3)	8 (3)	17 (6.5)	**<0.001**	46 (17.5)	29 (11)	26 (9.9)	43 (16.3)	40 (15.2)	79 (30.0)	**<0.001**	84 (31.9)	65 (24.7)	52 (19.8)	18 (6.8)	19 (7.2)	25 (9.5)	0.305	67 (25.5)	12 (4.6)	113 (43)	71 (27)	**0.028**
Body hair loss	4 (28.6)	6 (42.9)	2 (14.3)	--	2 (14.3)	0.636	2 (14.3)	2 (14.3)	2 (14.3)	1 (7.1)	1 (7.1)	6 (42.9)	0.443	3 (21.4)	8 (57.1)	1 (7.1)	--	2 (14.3)	--	0.14	3 (21.4)	1 (7.1)	1 (7.1)	9 (64.3)	**0.036**
Pruritus (itching)	45 (39.5)	47 (41.2)	12 (10.5)	5 (4.4)	5 (4.4)	0.654	14 (12.3)	19 (16.7)	13 (11.4)	24 (21.1)	15 (13.2)	29 (25.4)	0.361	29 (25.4)	34 (29.8)	26 (22.8)	11 (9.6)	11 (9.6)	3 (2.6)	0.307	46 (40.4)	10 (8.8)	25 (21.9)	33 (28.9)	**<0.001**
Gastroesophageal reflux	30 (34.5)	40 (46)	6 (6.9)	2 (2.3)	9 (10.3)	0.262	16 (18.4)	21 (24.1)	3 (3.4)	15 (17.2)	12 (13.8)	20 (23)	0.133	19 (21.8)	27 (31)	28 (32.2)	4 (4.6)	6 (6.9)	3 (3.4)	**0.019**	34 (39.1)	1 (1.1)	28 (32.2)	24 (27.6)	0.085
Skin dryness	63 (45.3)	46 (33.1)	15 (10.8)	3 (2.2)	12 (8.6)	0.378	23 (16.5)	17 (12.2)	19 (13.7)	32 (23)	22 (15.8)	26 (18.7)	0.132	31 (22.3)	46 (33.1)	27 (19.4)	15 (10.8)	16 (11.5)	4 (2.9)	**0.029**	50 (36)	9 (6.5)	33 (23.7)	47 (33.8)	**0.014**
Thrist	31 (40.3)	30 (39)	7 (9.1)	5 (6.5)	4 (5.2)	0.846	14 (18.2)	20 (26)	9 (11.7)	14 (18.2)	6 (7.8)	14 (18.2)	0.266	15 (19.5)	18 (23.4)	29 (37.7)	9 (11.7)	5 (6.5)	1 (1.3)	**<0.001**	34 (44.2)	3 (3.9)	20 (26)	20 (26)	**0.021**

Easy crying	79 (39.1)	80 (39.6)	26 (12.9)	3 (1.5)	14 (6.9)	0.062	35 (17.3)	29 (14.4)	30 (14.9)	33 (16.3)	24 (11.9)	51 (25.2)	0.113	46 (22.8)	62 (30.7)	53 (26.2)	18 (8.9)	15 (7.4)	8 (4)	**0.009**	75 (37.1)	12 (5.9)	60 (29.7)	55 (27.2)	**0.013**
Excessive sweating	41 (45.6)	32 (35.6)	8 (8.9)	4 (4.4)	5 (5.6)	0.872	11 (12.2)	16 (17.8)	10 (11.1)	23 (25.6)	9 (10)	21 (23.3)	0.316	28 (31.1)	29 (32.2)	14 (15.6)	6 (6.7)	9 (10)	4 (4.4)	0.709	25 (27.8)	2 (2.2)	37 (41.1)	26 (28.9)	0.512
Tremor of the extremities	33 (36.7)	40 (44.4)	7 (7.8)	4 (4.4)	6 (6.7)	0.734	15 (16.7)	11 (12.2)	13 (14.4)	13 (14.4)	13 (14.4)	25 (27.8)	0.251	18 (20)	31 (34.4)	21 (23.3)	8 (8.9)	8 (8.9)	4 (4.4)	0.218	28 (31.1)	11 (12.2)	25 (27.8)	26 (28.9)	**0.002**
Persistent cough	127 (51.8)	86 (35.1)	13 (5.3)	6 (2.4)	13 (5.3)	**<0.001**	70 (28.6)	57 (23.3)	28 (11.4)	40 (16.3)	21 (8.6)	29 (11.8)	**<0.001**	83 (33.9)	63 (25.7)	54 (22)	15 (6.1)	17 (6.9)	13 (5.3)	0.317	81 (33.1)	13 (5.3)	83 (33.9)	68 (27.8)	0.264
Brittle nails	56 (49.6)	36 (31.9)	12 (10.6)	4 (3.5)	5 (4.4)	0.225	18 (15.9)	13 (11.5)	21 (18.6)	24 (21.2)	15 (13.3)	22 (19.5)	**0.036**	34 (30.1)	29 (25.7)	24 (21.2)	6 (5.3)	15 (13.3)	5 (4.4)	0.411	39 (34.5)	5 (4.4)	28 (24.8)	41 (36.3)	0.092
Vomit	15 (60)	5 (20)	4 (16)	--	1 (4)	0.124	6 (24)	2 (8)	4 (16)	4 (16)	5 (20)	4 (16)	0.555	4 (16)	4 (16)	9 (36)	1 (4)	6 (24)	1 (4)	**0.017**	8 (32)	--	9 (36)	8 (32)	0.978
**Total**	**3257**	**2911**	**808**	**268**	**502**		**1297**	**1156**	**889**	**1503**	**1002**	**1899**		**2003**	**2284**	**1815**	**626**	**632**	**386**		**2486**	**433**	**2481**	**2346**	

Supplementary file S6. Distribution of long-COVID symptoms characteristics according to the characteristics of Ecuadorian participants.

**Characteristics**			**Sex n (%)**			**Comorbidities n (%)**			**Ethnicity n (%)**							**Vaccine**					
		**Total n**	**Male**	**Female**	**p Value**	**No**	**Yes**	**p Value**	**Afro**	**White**	**Indigenous**	**Mestizo**	**Montubio**	**Other**	**p Value**	**No**	**1 dose**	**2 doses**	**2 doses + 1 booster**	**2 doses + 2 boosters**	**p Value**
Onset time	Initiated with infection	449	148 (33.0)	301 (67.0)	0.093	359 (80.0)	90 (20.0)	0.452	3 (0.7)	27 (6.0)	6 (1.3)	406 (90.4)	5 (1.1)	2 (0.5)	0.382		2 (0.5)	68 (15.1)	323 (71.9)	56 (12.5)	0.753
	3 to 5 weeks after infection	414	153 (37.0)	261 (63.0)	0.654	320 (77.3)	94 (22.7)	0.409	2 (0.5)	26 (6.3)	1 (0.2)	382 (92.3)	2 (0.5)	1 (0.2)	0.198			63 (15.2)	296 (71.5)	55 (13.3)	0.906
	5 to 7 weeks after infection	103	33 (32.0)	70 (68.0)	0.44	83 (80.6)	20 (19.4)	0.721	1 (1.0)	1 (1.0)		98 (95.2)	3 (2.9)		0.088	1 (0.97)	1 (1.0)	15 (14.6)	69 (67.0)	17 (16.5)	**0.01**
	7 to 9 weeks after infection	50	26 (52.0)	24 (48.0)	**0.023**	41 (82.0)	9 (18.0)	0.688		1 (2.0)		46 (92.0)	2 (4.0)	1 (2.0)	0.056			15 (30.0)	32 (64.0)	3 (6.0)	**0.01**
	After 9 weeks of infection	84	36 (42.9)	48 (57.1)	0.213	63 (75.0)	21 (25.0)	0.465		4 (4.8)	1 (1.2)	76 (90.5)	3 (3.6)		0.314			12 (14.3)	61 (72.6)	11 (13.1)	0.926
Symptoms duration	Between 1 to 4 weeks	224	84 (37.5)	140 (62.5)	0.656	174 (77.7)	50 (22.3)	0.735	1 (0.5)	12 (5.4)	3 (1.3)	207 (92.4)	1 (0.5)		0.518			29 (13.0)	167 (74.6)	28 (12.5)	0.385
	Between 4 to 8 weeks	182	72 (39.6)	110 (60.4)	0.312	152 (83.5)	30 (16.5)	0.103	1 (0.6)	17 (9.3)	2 (1.1)	160 (87.9)	1 (0.6)	1 (0.6)	0.133	1 (0.55)		31 (17.0)	124 (68.1)	26 (14.3)	0.119
	Between 8 to 12 weeks	115	33 (28.7)	82 (71.3)	0.105	91 (79.1)	24 (20.9)	0.999		6 (5.2)		108 (93.9)	1 (0.9)		0.874			16 (13.9)	85 (73.9)	14 (12.2)	0.789
	Between 3 to 6 months	210	64 (30.5)	146 (69.5)	0.076	161 (76.7)	49 (23.3)	0.473	1 (0.5)	9 (4.3)		197 (93.8)	2 (1.0)	1 (0.5)	0.892		1 (0.5)	27 (12.9)	154 (73.3)	28 (13.3)	0.575
	Between 6 to 12 months	131	53 (40.5)	78 (59.5)	0.301	105 (80.2)	26 (19.9)	0.755		6 (4.6)	1 (0.8)	119 (90.8)	4 (3.1)	1 (0.8)	0.416			23 (17.6)	92 (70.2)	16 (12.2)	0.826
	More than 12 months	238	90 (37.8)	148 (62.2)	0.56	183 (76.9)	55 (23.1)	0.489	3 (1.3)	9 (3.8)	2 (0.8)	217 (91.2)	6 (2.5)	1 (0.4)	0.197		2 (0.8)	47 (19.8)	159 (66.8)	30 (12.6)	0.058
Frequency of presentation	Once a month	97	40 (41.2)	57 (58.8)	0.31	76 (78.4)	21 (21.7)	0.999		4 (4.1)		91 (93.8)	2 (2.1)		0.704			15 (15.5)	68 (70.1)	14 (14.4)	0.902
	Once every two weeks	84	30 (35.7)	54 (64.3)	0.999	70 (83.3)	14 (16.7)	0.349	1 (1.2)	6 (7.1)	1 (1.2)	75 (89.3)	1 (1.2)		0.815		1 (1.2)	12 (14.3)	60 (71.4)	11 (13.1)	0.402
	Once a week	216	80 (37.0)	136 (63.0)	0.783	180 (83.3)	36 (16.7)	0.079		12 (5.6)	2 (0.9)	198 (91.7)	3 (1.4)	1 (0.5)	0.994		1 (0.5)	32 (14.8)	164 (75.9)	19 (8.8)	0.177
	Over 3 days a week	297	108 (36.4)	189 (63.6)	0.935	234 (78.8)	63 (21.2)	0.999	3 (1.0)	13 (4.4)		273 (91.9)	5 (1.7)	3 (1.0)	0.122			55 (18.5)	203 (68.4)	39 (13.1)	0.297
	Daily	331	110 (33.2)	221 (66.8)	0.235	242 (73.1)	89 (26.9)	**0.003**	2 (0.6)	19 (5.7)	2 (0.6)	304 (91.8)	4 (1.2)		0.988	1 (0.3)		47 (14.2)	236 (71.3)	47 (14.2)	0.304
	No apply	75	28 (37.3)	47 (62.7)	0.901	64 (85.3)	11 (14.7)	0.193		5 (6.7)	3 (4.0)	67 (89.3)			**0.002**		1 (1.3)	12 (16.0)	50 (66.7)	12 (16.0)	0.248

**Total**	**3300**	**1188**	**2112**	**2598**	**702**	**18**	**177**	**24**	**3024**	**45**	**12**	**3**	**9**	**519**	**2343**	**426**

Supplementary file S7. Risk of long-COVID symptoms lasting more than 6 months according to the number of COVID-19 infections.

		**1 Infection**		**> 1 Infection**		**RR**	**Probability**	**P value**	**95% CI**
		**n**	**(%)**	**n**	**(%)**				
**Symptoms duration**	< 6 Months *(ref.)*	576	69.6%	155	56.8%				
	> 6 Months	251	30.4%	118	43.2%	1.296	29.6%	0.006	**1.076 - 1.560**
	Total	827	100.0%	273	100.0%	N/A		N/A	

RR: relative risk

Supplementary file S8. Risk of long-COVID symptoms lasting longer than 12 months according to the number of COVID-19 infections.

		**1 Infection**		**> 1 Infection**		**RR**	**Probability**	**P value**	**95% CI**
		**n**	**(%)**	**n**	**(%)**				
**Symptoms duration**	1 Month *(ref.)*	187	22.6%	37	13.6%	0.647	-35.3%	0.009	0.4658 - 0.8993
	2-3 Months *(ref.)*	224	27.1%	73	26.7%	0.989	-1.0%	0.085	0.7830 - 1.2516
	3-11 Months *(ref.)*	254	30.7%	87	31.9%	1.028	2.8%	0.795	0.8318 - 1.2717
	> 12 Months	162	19.6%	76	27.8%	1.329	32.9%	0.022	**1.0420 - 1.6962**
	Total	827	100.0%	273	100.0%	N/A		N/A	

RR: relative risk
